# Immune‐enriched phyllosphere microbiome in rice panicle exhibits protective effects against rice blast and rice false smut diseases

**DOI:** 10.1002/imt2.223

**Published:** 2024-07-15

**Authors:** Dacheng Wang, Yingqiao Wan, Dekun Liu, Ning Wang, Jingni Wu, Qin Gu, Huijun Wu, Xuewen Gao, Yiming Wang

**Affiliations:** ^1^ Department of Plant Pathology, Key Laboratory of Integrated Management of Crop Diseases and Pests, Ministry of Education Nanjing Agricultural University Nanjing China; ^2^ Research Center for Functional Microbiology Organic Recycling Research Institute (Suzhou) of China Agricultural University Suzhou China

## Abstract

Activation of immune responses leads to an enrichment of beneficial microbes in rice panicle. We therefore selected the enriched operational taxonomy units (OTUs) exhibiting direct suppression effects on fungal pathogens, and established a simplified synthetic community (SynCom) which consists of three beneficial microbes. Application of this SynCom exhibits protective effect against fungal pathogen infection in rice.

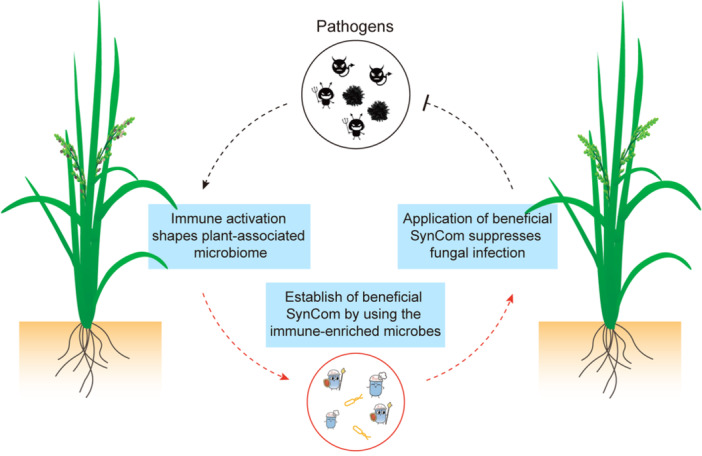

Beneficial microbes exhibit positive effects on health not only in plants but also in animals and humans [1, 2, 3], which give possibility of their utilization for improving crop health under adverse environmental conditions [4, 5], disease resistance [6, 7, 8], and nutrient acquisition [9, 10]. A recent study reveals that infection of *Fusarium oxysporum* leads to an alternation of bacterial and fungal communities in chili pepper which showed an enrichment of beneficial bacteria during infection [11]. These beneficial microbes play a crucial role in the activation of plant immunity against diverse pathogens. Notably, the activation of immunity is always accompanied by reduced plant growth due to growth‐defense trade‐off [12]. However, the plant‐associated microbes could buffer the microbe‐associated molecular pattern (MAMP)‐triggered growth suppression and thus maintain the plant growth [13]. Therefore, the identification and characterization of plant‐associated beneficial microbes may offer a potential strategy for their utilization in agricultural practices in a sustainable manner.

The microbial communities in each rice compartment have been investigated to understand their composition and diversity [14]. The rhizosphere and phyllosphere are the compartments where plants directly interact with environments. Microbiomes in these compartments are more diverse due to different environmental conditions. In contrast, the endosphere microbes exist in the internal regions of plant tissues with relatively lower abundance. Interestingly, operational taxonomy units (OTUs) in endosphere may be transmitted to the next generation through seeds [14]. Therefore, beneficial endophytes are considered as biocontrol agents which may protect plants over generations. However, detection of plant endosphere microbes is relatively challenging due to the vast amount of host genomic DNA that imposes technical limitations in the isolation and characterization of microbial DNA. Furthermore, the biological functions of endophytes are still not well illustrated.

In this study, we analyzed the microbiome composition in rice panicle at the booting stage after challenging two major fungal pathogens. Significant alterations of microbiome composition were detected compared with the untreated plants, suggesting that pathogen infection alternates rice endophytes. To investigate the biological function of those pathogen‐induced microbes, 11 OTUs belonging to seven species were isolated of which six could directly suppress the *Magnaporthe grisea* and *Ustilaginoidea virens* in vitro. A simplified synthetic community (SynCom) consisting of three OTUs belonging to *Pantoea agglomerans*, *Acidovorax wautersii*, and *Burkholderia pyrrocinia* was established. Application of this SynCom exhibited a protective role in rice against *M. oryzae* and *U. virens* under both laboratory and field conditions. Our findings thus provide evidence that pathogen‐induced endosphere microbes exhibit beneficial effects on rice and offer a new strategy for the development of biocontrol agents for maintaining rice productivity.

## RESULTS

### Disease‐induced alteration of perithecium‐associated bacterial communities

**Figure 1:**
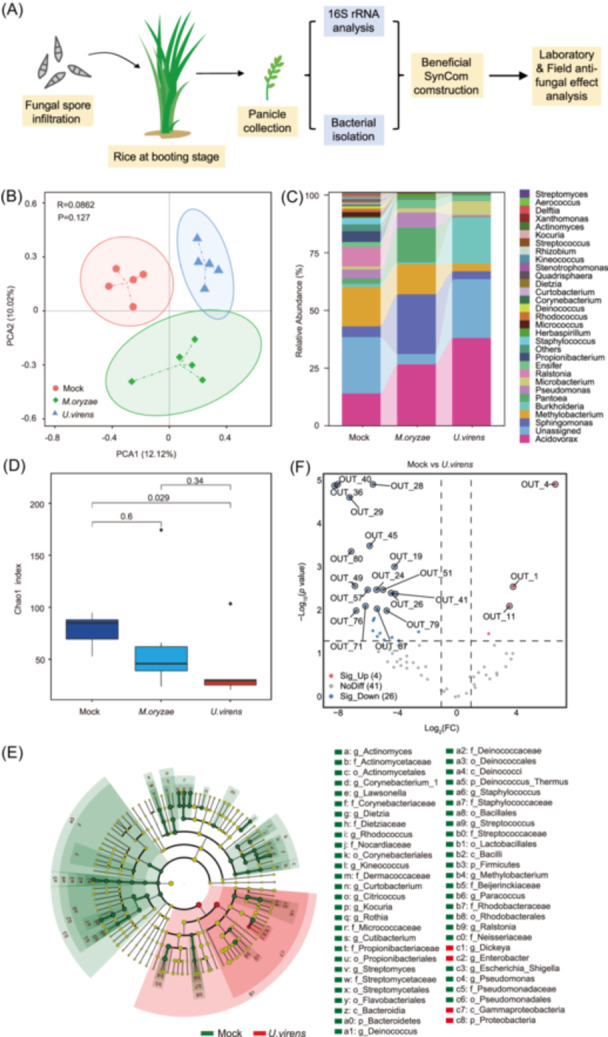
Analysis of panicle microbiome in pathogen‐infected rice plants. (A) Pipeline for high‐throughput bacterial cultivation and identification. (B) Principal component analysis (PCA) analysis of bacterial communities based on the Bray‐Curtis distances calculated from the relative abundance of different operational taxonomy units (OTUs). (C) Changes in the panicle microbiome composition at the genera level. (D) A comparison of bacterial community diversity between the panicles of pathogens infected and noninfected rice plants was implemented by analysis of Chao1 index. Values are means ± SD (five biological replicates, each including a mixture of six panicles), and *p*‐values are shown above the paired columns according to unpaired Student's *t*‐test (two‐tailed). (E) The taxonomic cladogram resulting from LEfSe analysis of 16S rRNA sequences highlights biomarkers linked to treatments for *Ustilaginoidea virens*. Only taxa surpassing an linear discriminant analysis (LDA) significance threshold of 3.5 are depicted, with their abundance within each group represented by small circles and varying shades in the diagram. Yellow circles signify insignificant differences in abundance between treatments within specific taxonomic groups. The brightness of each dot corresponds to its effect size. Taxa enriched in the control group are shown with a negative LDA score (green), while those enriched in *U. virens* are indicated with positive scores (red). (F) The volcano plot displays differentially accumulated OTUs in the panicle following and *U. virens* infection. Each point represents an individual OTU, and the position along the *x*‐axis represents the abundance of fold change (FC). The dashed lines show the threshold of significant differential OTUs (|log_2_ (FC)| > 1). Blue dots, red dots, and gray dots represent significant enriched OTUs (up), significant depleted OTUs (down), and OTUs with no difference, respectively.

Fungal diseases in rice panicle strongly affect rice productivity. Since the potential beneficial bacteria could be enriched during pathogen infection, we investigated the phyllosphere microbiome dynamics in the rice panicle at the booting stage in response to rice blast and rice false smut diseases. The phyllosphere microbial profile was analyzed at 48 h postinfiltration of either the sterilized water (Mock) or spore suspensions of *M. oryzae* and *U. virens* (Figure 1A). At this point, no disease symptoms occurred, but significant immunity took place in the panicle without disease symptoms. We therefore hypothesize that the microbiome is alternated by rice immunity rather than the occurrence of diseases. A total of 141,997 high‐quality reads were obtained, which were assigned to 282 bacterial OTUs after the removal of OTUs taxonomically classified as mitochondria or chloroplasts. The 16S rRNA sequences were taxonomically assigned to six bacterial phyla, encompassing 51 genera and 137 OTUs (Table S1). Unconstrained principal coordinate analysis revealed that the panicle bacterial communities formed distinct clusters in mock and fungal‐infected panicles (Figure 1B). Taxonomic analysis found that the rice panicle bacteria comprised mainly of two phyla (Figure S1, Table S1), among which *Proteobacteria* was the most abundant (95.01%), followed by *Actinobacteria* (4.62%). The relative abundance of the top 30 most abundant genera was highlighted, revealing other dominant genera belonging to *Acidovorax*, *Sphingomonas*, *Methylobacterium*, and *Burkholderia* (Figure 1C). Of these, the relative abundance of particularly *Acidovorax* was increased in both *M. oryzae* and *U. virens*‐infected rice panicles, while the *Methylobacterium* was reduced. Besides, the relative abundance of *Sphingomonas* was interestingly increased in *M. oryzae‐*infected rice panicles and decreased in *U. virens*‐infected samples while *Burkholderia* showed a reverse trend. The alpha‐diversity assessment revealed that pathogen infections showed a trend for a reduction of fungal diversity, and *U. virens* exhibits stronger effect than that of *M. oryzae* (Figure 1D, Figure S2A), highlighting a reduced microbiome diversity and increased microbial richness.

### Biomarker taxa of bacterial microbiome among different disease infection

To further understand which microbes were remarkably altered upon fungal infection, a linear discriminant analysis (LDA) effect size (LEfSe) analysis was performed, which demonstrated that *Pantoea* and *Sphingomonas* were specifically enriched in response to *M. oryzae* (Figure S2B), while *Dickeya* and *Enterobacter* were selectively enriched in *U. virens*‐infected panicles (Figure 1E). The volcano plot analysis displayed the differentially accumulated OTUs in panicle upon infection of two different fungal diseases. Five OTUs belonging to *Acidovorax*, *Pantoea*, *Pseudomonas*, *Sphingomonas*, and *Herbaspirilum* were highly enriched, whereas 11 OTUs were depleted upon *M. oryzae* infection compared with that of mock (Figure S2C). In response to *U. virens*, three OTUs (OTU1, OTU4, and OTU11) belonging to *Acidovorax*, *Burkholderia*, and *Aurantimonadaceae* were enriched while 17 OTUs were reduced (Figure 1F).

### Bioassay‐directed screening affords strain‐inhibiting rice pathogens

**Figure 2:**
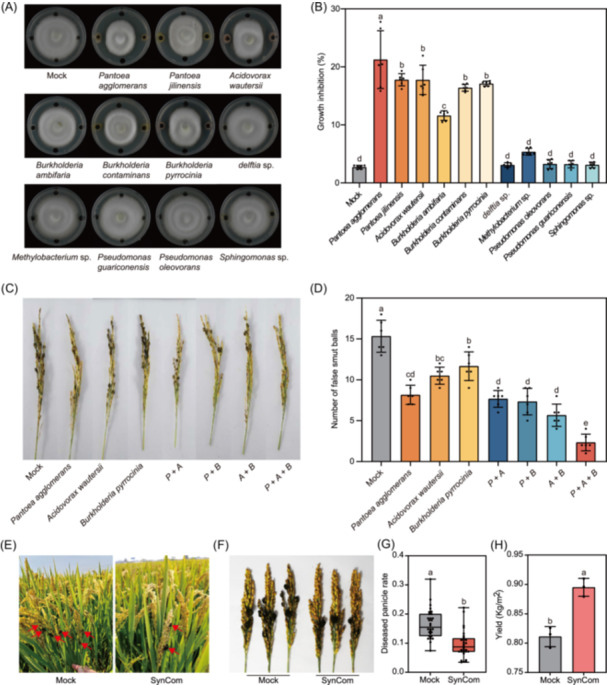
Antagonistic and biocontrol activity of identified bacterial strain inhibiting rice fungal pathogen *Ustilaginoidea virens*. (A) Antagonistic activity of screening strain toward *U. virens* in cocultured assays, using untreated strain as control (mock). (B) The growth of inhibition of screening strain toward *U. virens.* The experiments were repeated three times independently with similar results. (C) Biocontrol activity of screening strain toward *U. virens.* (D) The number of *U. virens* false smut balls was altered in rice panicles treated with different antagonistic isolates. Values are means ± SD (*n* = 6 panicles). Different letters indicate significant differences at *p* < 0.05, as determined by one‐way analysis of variance with Tukey's multiple comparisons test. The experiments were repeated three times independently with similar results. (E) Field test of the disease suppression activity of the SynCom (P + A + B). (F) Representative images of *U. virens* symptoms in rice panicles. (G) The diseased panicle rate treated with the SynCom. Values are means ± SD (*n* = 30). Different letters indicate significant differences at *p* < 0.05, as determined by unpaired Student's *t*‐test (two‐tailed). (H) Statistics of rice yield after SynCom treatment. Different letters indicate significant differences at *p* < 0.01, as determined by unpaired Student's *t*‐test (two‐tailed).

We hypothesize that rice could also recruit beneficial bacteria for countering the infection of pathogens. Among 189 cultivable bacterial isolates obtained from panicle samples, we selected 11 microbes belonging to *Pantoea* (OTU6), *Acidovorax* (OTU1), *Burkholderia* (OTU4), *Delftia* (OTU76), *Methylobacterium* (OTU26), *Pseudomonas* (OTU8), and *Sphingomonas* (OTU15) (Figure S3), which were belonging to the differentially accumulated taxa in either *M. oryzae* or *U. virens*‐infected panicles for the in vitro growth suppression assay. Among these, the *P. agglomerans*, *P. jilinensis*, *A. wautersii*, *B. contaminans*, and *B. pyrrocinia* exhibited strongest antagonistic effects on *M. oryzae* (Figure S4A,B) and *U. virens* growth (Figure 2A,B). In contrast, the OTUs depleted upon fungal infection did not show growth inhibition effects (Figure 2A,B).

### Construction of a simplified disease‐resistant community

Three isolates exhibiting pathogen suppression effects including *P. agglomerans*, *A. wautersii*, and *B. pyrrocinia* were used for establishing a SynCom. These SynCom microbes were leaf sprayed on rice seedlings in individual, paired, or mixed manner followed by inoculation of *M. oryzae* at 24 h post‐SynCom treatment. Intriguingly, individual OTUs slightly enhanced rice resistance, whereas combinations of two OTUs increased *M. oryzae* resistance more significantly. The SynCom (P + A + B) consisting of *P. agglomerans*, *A. wautersii*, and *B. pyrrocinia* exhibited the most efficient effect on *M. oryzae* resistance (Figure S4C,D). Similarly, the SynCom (P + A + B) exhibited a much stronger suppression effect on the rice false smut balls formation as compared to individually or in a combination of two (paired) strains (Figure 2C,D).

Filed test was further performed by spraying of SynCom (P + A + B) on rice at seedling transplanting, tillering stage, and between booting and heading stages. As compared to the untreated plants, SynCom treatment enhances rice resistance against both *M. oryzae* (Figure S4E–G) and *U. virens* (Figure 2E–G), which leads to an increase of rice productivity (Figure 2H). Taken together, our data showed that SynCom exhibits defense‐enhancing effects on rice cultivars and can be sustainably used for durable protection.

## DISCUSSION

Plants are continuously exposed to the pathogenic microbes that tend to limit their growth. Plants employ a multilayered immune system to counter against the pathogenic microbes. Additionally, accumulating evidence suggests that plants could also employ beneficial microbes to fight against pathogenic infections. However, such information on rice is currently limited. In this study, we hypothesized that the fungal infection induces activation of plant immunity, which may cause alternation in the rice microbiome, resulting in the enrichment of beneficial microbes that subsequently enhance disease resistance. We therefore investigated the changes in microbiome dynamics and functions of individual microbes in the suppression of pathogenic fungi. Furthermore, by using a simplified beneficial SynCom, their protective effects against both rice blast and rice false smut diseases were validated by indoor and in the field tests. Taken together, we demonstrated that the disease‐enriched beneficial microbes exhibit beneficial effects on rice disease resistance through direct suppression of fungal growth.

Plenty of microbiome studies have been performed to investigate the microbiome composition in different tissues of rice plants. Compared to the root rhizosphere and root, the microbiome diversity and abundance were reported to be lesser in the seed endosphere [15]. The core microbiome taxa in the seed endosphere are vertically transmitted which is probably important for the improvement of rice fitness [15]. Here, we also noticed that the OTUs were also in a relatively low abundance with a lesser diversity. It may be possible that the microbiome samples harvested from panicles at the booting stage are tightly covered by the stem tissues. We therefore analyzed the microbiome in the panicle after heading, in which the panicles were exposed to natural conditions. Principal component analysis analysis showed a significant diversity of microbes in the panicle after heading, and the distance was even larger than the effect observed by the fungal infection (Figure S5).

Compared to the microbe composition, the *Betaproteobacteria* and *Bacilli* in the rice seeds were relatively lesser [15]. Here, the composition of *Betaproteobacteria* was the highest; however, *Bacilli* was not detected. Moreover, from our microbiome cultures, none of *Bacilli* was successfully isolated. It is known that *Bacillus* strains are frequently used as bioprotective agents for disease resistance in plants [16]. Recently, we also reported that the rice root‐associated *Bacillus* enhances rice resistance against *Xanthomonas* infection [17]. Since the panicle is a reproductive tissue of rice, the panicle cells may have a relatively lower immunity due to the growth‐defense tradeoff. Therefore, it is possible that microbes exhibiting direct inhibitory effects on pathogens are recruited with priority. Besides, it may also be possible that the strategies for beneficial microbe recruitment in rice panicles may be different than other tissues such as root and leaf. In particular, the *A. wautersii* exhibited a direct inhibitory effect on the mycelial growth of *M. oryzae* and *U. virens* while the depleted OTUs did not display any suppression effects (Figure 2, Figure S3). Moreover, exogenous application of *A. wautersii* also showed attenuation of fungal infection on rice (Figure 2, Figure S3). This is consistent with the previous findings that disease‐induced assembly of beneficial microbes, highly enriched in the rhizosphere, increases plant disease resistance [11, 18]. Therefore, rice may employ different mechanisms for resistance against different types of fungal pathogens.

## METHODS

Detailed procedures for biological sample collection, bacterial isolation, sequencing protocol, data processing techniques for sequencing data, and bioinformatic and statistical analysis approaches are available in the Supplementary Information.

## CONCLUSION

In conclusion, our study uncovers that fungal infection by *M. oryzae* and *U. virens* leads to an enrichment of beneficial phyllosphere microbes in the rice panicle. We next examined the effects of enriched beneficial microbes and developed a simplified SynCom, consisting of three beneficial bacteria, that was able to inhibit the mycelia growth of fungal pathogens directly. Notably, the application of SynCom significantly enhanced rice resistance to *M. oryzae* and *U. virens* in both laboratory and field conditions. In essence, our results suggest that the beneficial microbes, especially pathogen infection‐enriched microbes, can be efficiently utilized for the management of deadly plant diseases for sustainable agriculture practices in a cost‐effective manner.

## AUTHOR CONTRIBUTIONS

Yiming Wang conceptualized the research program, designed the experiments, and coordinated the project. Dacheng Wang, Yingqiao Wan, Jingni Wu, Qin Gu, Dekun Liu, Ning Wang, Huijun Wu, and Xuewen Gao performed the experiment and analyzed the data. Dacheng Wang and Yiming Wang wrote the manuscript. All authors have read the final manuscript and approved it for publication.

## CONFLICT OF INTEREST STATEMENT

The authors declare no conflict of interest.

## ETHICS STATEMENT

No animals or humans were involved in this study.

## Supporting information


**Figure S1:** Changes in the panicle microbiome composition at the phylum level.
**Figure S2:** Analysis of panicle microbiome in *M. oryzae*‐infected rice plants.
**Figure S3:** Molecular Phylogenetic analysis by Maximum Likelihood method.
**Figure S4:** Antagonistic and biocontrol activity of identified bacterial strain inhibiting rice fungal pathogen *M. oryzae*.
**Figure S5:** Principal Component Analysis (PCA) analysis of bacterial communities.

**Table S1:** Taxonomic annotation of OTUs.
**Table S2:** ASV abundance of OTUs.

## Data Availability

The data that support the findings of this study are openly available in PRJCA024733 at https://ngdc.cncb.ac.cn/, reference number AMC3481738, SAMC3481739, SAMC3481740, SAMC3481741, and SAMC34817. All the sequencing data have been deposited in the National Genomics Data Center under submission number subPRO036692 and BioProject accession number PRJCA024733 (https://ngdc.cncb.ac.cn/gsub/submit/bioproject/subPRO036692/overview). The data and scripts used have been saved in GitHub https://github.com/bossning/Immune-induced-rice-panicle-microbiome-alternation. Supporting Information (methods, figures, tables, graphical abstract, slides, videos, Chinese translated version, and updated materials) can be found in the online DOI or iMeta Science http://www.imeta.science/.
