# Supplementary material for: Immune‐enriched phyllosphere microbiome in rice panicle exhibits protective effects against rice blast and rice false smut diseases
Source: Imeta. 2024 Jul 15;3(4):e223. doi: 10.1002/imt2.223 (PMC11316918; doi:10.1002/imt2.223)
Supplement: Supplementary file 1 — Figure S1: Changes in the panicle microbiome composition at the phylum level. Figure S2: Analysis of panicle microbiome in M. oryzae‐infected rice plants. Figure S3: Molecular Phylogenetic analysis by Maximum Likelihood method. Figure S4: Antagonistic and biocontrol activity of identified bacterial strain inhibiting rice fungal pathogen M. oryzae. Figure S5: Principal Component Analysis (PCA) analysis of bacterial communities. [file IMT2-3-e223-s001.docx]

**Supplementing information to**

**Immune-enriched phyllosphere microbiome in rice panicle exhibits protective effects against rice blast and rice false smut diseases**

**Runing title:** Immune-induced rice panicle microbiome alternation

Dacheng Wang^1#^, Yingqiao Wan^1#^, Dekun Liu^1#^, Ning Wang^2^, Jingni Wu^1^, Qin Gu^1^, Huijun Wu^1^, Xuewen Gao^1^, Yiming Wang^1*^

^1^Department of Plant Pathology, Key Laboratory of Integrated Management of Crop Diseases and Pests, Ministry of Education, Nanjing Agricultural University, Nanjing 210095, China.

^2^Research Center for Functional Microbiology, Organic Recycling Research Institute (Suzhou) of China Agricultural University, Suzhou 215168, China.

^#^These authors contributed equally: Dacheng Wang, Yingqiao Wan, Dekun Liu.

^*^Correspondence: [ymwang@njau.edu.cn](mailto:mywang@njau.edu.cn) (Yiming Wang).

**Supplementary materials and methods**

**Sample collection**

All microbiome samples were collected from rice panicles 24 h after pathogen inoculation at the booting stage, cultivated at the Baima Teaching and Research base of Nanjing Agricultural University (31°61’32” N, 119°17’87” E), Nanjing, China. Field test was performed in Jurong (31°77’58” N, 119°20’79” E), Jiangsu, China.

**Plant material, bacterial and fungal culture conditions**

The rice (*Oryza sativa* L. *japonica* cv. Zhonghua 11) was used in this study. The rice cultivars (*O. sativa* cv. Nangeng 46) used for field tests were the same as those planted by local farmers. Each treatment included five replicates. Isolated bacterial strains from rice panicles were serially diluted and then cultured on Luria-Bertani (LB) plate at 28 ℃ for 24 h. A single colony from a freshly streaked plate was picked and inoculated into LB broth and incubated at 28 ℃ for 16 h. Bacterial cells were subsequently collected by centrifugation, washed twice with sterile water, and re-suspended in sterile water to a final density of ~0.5 OD_600_ for inoculation_._ *M. oryzae* TH12, used in this study, was cultured in a CM medium. For conidiation, strain blocks were maintained on a straw decoction and corn agar medium at 28 °C for 7 days in the dark, followed by 3 days of continuous illumination under fluorescent light. Conidia were resuspended to a concentration of 1×10^5^ spores/mL in a 0.2% (w/v) gelatin solution for further infection. *U. virens* HWD, used in this study was cultured on potato‐sucrose‐agar medium, transferred into potato‐sucrose broth (PSB), and incubated at 28 °C and 120 rpm for 5 days. The blended mixture of mycelia and conidia was adjusted to 1 × 10^6^ conidia/ml for further infection.

**DNA extraction and amplicon sequencing**

The panicles were surface sterilized with 70% ethanol and then with 2% sodium hypochlorite. Each replicate included four panicles. The panicles were ground into fine powder in liquid nitrogen using a homogenizer (Shanghai Jingxin). Total microbial community DNA was extracted using a Fast DNA spin Kit for soil (MP, Biomedicals). The quality of all DNA samples was detected by NanoDrop spectrophotometer (Thermo Scientific). The extracted DNAs were stored at -20 °C until analysis. The bacterial 16S rRNA gene fragments were amplified with the universal primers 799 F (5′-AACMGGATTAGATACCCKG-3′) and 1193 R (5′-ACGTCATCCCCACCTTCC-3′) fused with unique barcode [1]. Gel-purified polymerase chain reaction products were mixed in an equal molar concentration following illumine sequencing using the platform Hiseq 2500.

**Bioinformatic and statistical analysis**

The sequences obtained were demultiplexed and filtered for quality using Vsearch (version v2.0.3) [2]. Paired-end reads were assessed using FastQC (version 0.10. 136) [3] and merged using the Vsearch script fastq_mergepairs command. After removing barcodes and primers, low-quality reads were discarded, and non-redundant reads were identified. The zero-radius operational taxonomy units (OTUs) (zOTUs/OTU) table was constructed using UNOISE3. After eliminating OTUs that were not detected in at least 1% of all samples, a total of 1,528,160 raw reads were identified with an average length of 394 bp across 15 samples. The taxonomic classification of each sequence was performed using the RDP Classifier against the SILVA small subunit rRNA database (version 138) with a confidence threshold of 0.7, following quality trimming [4,5]. The generated sequencing data (raw fastq files) was submitted to the National Genomics Data Center BioProject database (https://ngdc.cncb.ac.cn/) (BioProject accessions: PRJCA024733; BioSample accessions: AMC3481738, SAMC3481739, SAMC3481740, SAMC3481741, SAMC3481742, SAMC3481743, SAMC3481744, SAMC3481745, SAMC3481746, SAMC3481747, SAMC3481748, SAMC3481749, SAMC3481750, SAMC3481751, SAMC3481752, SAMC3481753, SAMC3481754, SAMC3481755, SAMC3481756, SAMC3481757).

**Bacterial isolation, identification, and assessment of antifungal activity**

The panicles were sequentially surface sterilized with 70% ethanol and 2% sodium hypochlorite. Sterilized panicles were homogenized in phosphate-buffered saline with Tween-20 detergent (PBST) buffer (Na_2_HPO_4_ 1.42 g/L, KH_2_PO_4_ 0.24 g/L, NaCl 8 g/L, KCl 0.2 g/L, 0.01% Tween-20, pH 7.4) with shaking (180 rpm, 30 °C) for 1 h. Homogenized samples were then allowed to settle for 20 min and the supernatants were serially diluted. The resulting supernatants were plated and cultivated on 9 cm Petri dishes in 1:10 (v/v) tryptic soy (TSB) medium, Reasoner’s 2A agar medium, and LB medium for 3d at 30 °C. All bacteria were isolated by picking colonies. The antagonistic activity of bacterial isolates towards *M. oryzae* and *U. virens* was assessed with a dual-culture assay in PSA agar. The *M. oryzae* and *U. virens* were cultured on PSA agar medium for 2 days and 5 days, respectively. Isolated bacterial strains were dropped on the growth medium. All isolates were tested in triplicates, and their inhibition zones were measured after 4-5 days and 7 days of dual-culture cultivation at 25 °C for *M. oryzae* and *U. virens*, respectively. The antagonistic isolates were identified by sequencing the 16S rDNA housekeeping *gyrB* gene and the sequences were compared by using the basic local alignment search tool (BLAST), with the reference sequences in the Nucleotide Sequence Database of NCBI (National Center for Biotechnology Information).

***In vivo* infection assays**

Germinated rice seeds were grown in MS solid medium for 7 d and then transferred onto sterilized soil. The seedlings were transplanted in pots and grown in a glass greenhouse. For biocontrol experiments, rice seedlings were root inoculated with the isolated strains by pipetting 10 ml of ~0.5 OD_600_ aqueous bacterial suspension. *M. oryzae* and *U. virens* were cultivated as described previously. The 15-day-old seedlings were used for *M. oryzae* infection. A conidial suspension was sprayed onto seedlings. Inoculated plants were kept in a growth chamber at 25 ℃ with 90% humidity and in the dark for the first 24 h, followed by a 12 h/12 h light/dark cycle for 7 days. For *U. virens* infection, a 1 mL mixture of mycelium and spore suspension was injected into rice panicles of late booting stages seedlings (5–7 days before heading).

**Statistics analysis**

All statistical analyses were conducted using IBM SPSS Statistics software for Windows (Version 21.0, Armonk, NY, USA) and R software (Version 3.6.3, http://www.R-project.org, R base, and Vegan package). One-way ANOVA was utilized to determine significant differences (*p* < 0.05). Principal component analysis (PCA) was employed to visualize community composition, with pairwise Bray-Curtis distances calculated from the relative abundance of different OTUs. Richness (Chao1) and diversity (Shannon) indexes were assessed at the OTU level, with abundance and diversity estimates performed in R. The study employed the linear discriminant analysis effect size (LEfSe, http://huttenhower.sph.harvard.edu/galaxy) test to unveil significant differences in bacterial taxa with relative abundance above 0.01% between treatments [6]. In LEfSe analysis, the Kruskal-Wallis (KW) sum-rank test identified features with substantially different abundances between assigned classes, while linear discriminant analysis (LDA) quantified the effect size of each differentially abundant taxon, adhering to an effect-size threshold of 2 for all biomarkers [7,8].

**REFERENCES**

1. Beckers, Bram, Michiel Op De Beeck, Sofie Thijs, Sascha Truyens, Nele Weyens, Wout Boerjan, and Jaco Vangronsveld. 2016. “Performance of 16s rDNA primer pairs in the study of rhizosphere and endosphere bacterial microbiomes in metabarcoding studies.” *Frontiers in microbiology*. 7, 177245. <http://10.3389/fmicb.2016.00650>
2. Rognes, Torbjørn, Tomáš Flouri, Ben Nichols, Christopher Quince, and Frédéric Mahé. 2016. “VSEARCH: a versatile open source tool for metagenomics.” *PeerJ*. 4, e2584. <http://doi.10.7717/peerj.2584>
3. Brown, Joseph, Meg Pirrung, and Lee Ann McCue. 2017. “FQC Dashboard: integrates FastQC results into a web-based, interactive, and extensible FASTQ quality control tool.” *Bioinformatics.* 33(19), 3137-3139. <http://10.1093/bioinformatics/btx373>
4. Wang, Qiong, George M Garrity, James M Tiedje, and James R Cole. 2007. “Naive Bayesian classifier for rapid assignment of rRNA sequences into the new bacterial taxonomy.” *Applied and environmental microbiology*. 73(16), 5261-5267. <http://doi.10.1128/AEM.00062-07>
5. Quast, Christian, Elmar Pruesse, Pelin Yilmaz, Jan Gerken, Timmy Schweer, Pablo Yarza, Jörg Peplies, and Frank Oliver Glöckner. 2012. “The SILVA ribosomal RNA gene database project: improved data processing and web-based tools.” *Nucleic acids research.* 41(D1), D590-D596. <http://doi.10.1093/nar/gks1219>
6. Tao, Chengyuan, Rong Li, Xiong Wu, Zongzhuan Shen, Shanshan Liu, Beibei Wang, Yunze Ruan, Stefan Geisen, Qirong Shen, and George A Kowalchuk. 2020. “Bio-organic fertilizers stimulate indigenous soil *Pseudomonas* populations to enhance plant disease suppression.” *Microbiome.* 8, 1-14. <http://doi.10.1186/s40168-020-00892-z>
7. Segata, Nicola, Jacques Izard, Levi Waldron, Dirk Gevers, Larisa Miropolsky, Wendy S Garrett, and Curtis Huttenhower. 2011. “Metagenomic biomarker discovery and explanation.” *Genome biology*. 12, 1-18. <http://doi.10.1186/gb-2011-12-6-r60>
8. Tamura, Koichiro and Masatoshi Nei. 1993. “Estimation of the number of nucleotide substitutions in the control region of mitochondrial DNA in humans and chimpanzees.” *Molecular Biology and Evolution.* 10,512-526. [http://doi.10.1093/oxfordjournals.molbev.a040023](http://doi.10.1186/gb-2011-12-6-r60)

**Supplementary figures**

**
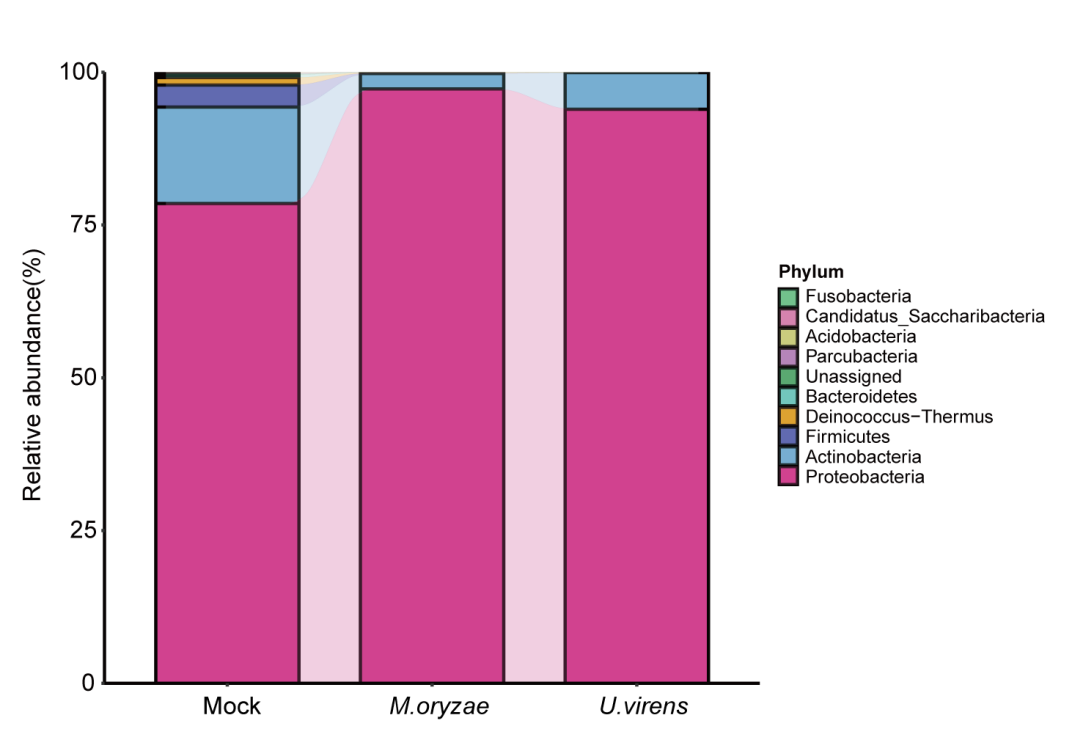
**

**Figure S1 Changes in the panicle microbiome composition at the phylum level.**

**
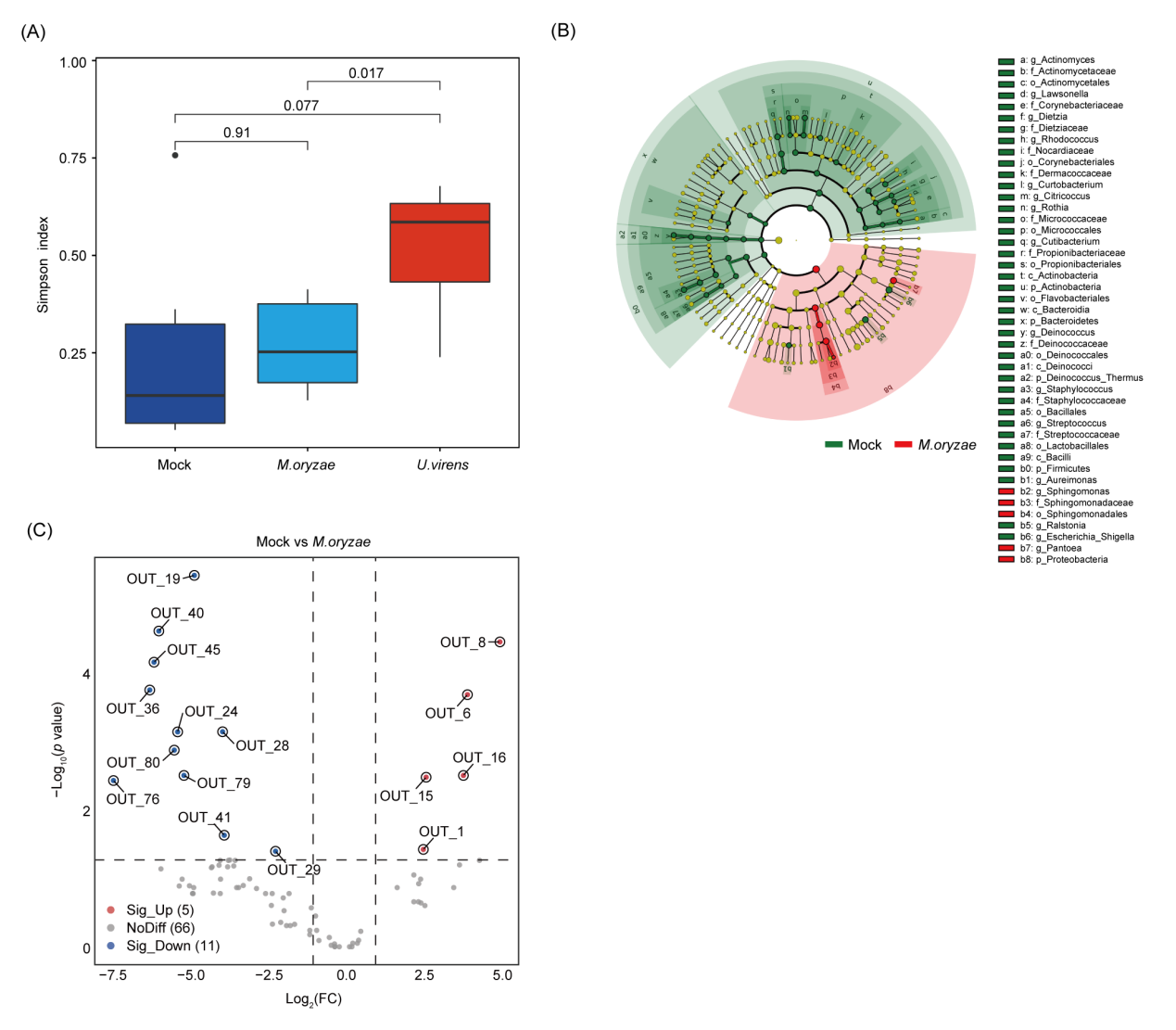
**

**Figure S2 Analysis of panicle microbiome in *M. oryzae*-infected rice plants.** (A) A comparison of bacterial community diversity between the panicles of *M. oryzae* infected and non-infected rice plants was implemented by analysis of Simpson index. Values are means ± SD (n = 5 biological replicates, each including a mixture of 6 panicles) and *p*-values are shown above the paired columns according to unpaired Student’s t-test (two-tailed). (B) The taxonomic cladogram resulting from LEfSe analysis of 16S rRNA sequences highlights biomarkers linked to treatments for *M. oryzae*. Only taxa surpassing an LDA significance threshold of 3.5 are depicted, with their abundance within each group represented by small circles and varying shades in the diagram. Yellow circles signify insignificant differences in abundance between treatments within specific taxonomic groups. The brightness of each dot corresponds to its effect size. Taxa enriched in the control group are shown with a negative LDA score (green), while those enriched in *M. oryzae* are indicated with positive scores (red). (F) The volcano plot displays differentially accumulated OTUs in the panicle following and *M. oryzae* infection. Each point represents an individual OTU and the position along the x-axis represents the abundance fold change. The dashed line showed the threshold of significant differential OTUs (|log_2_ (FC)| > 1). Blue dots, red dots, and grey dots represent significant enriched OTUs (up), significant depleted OTUs (down), and no difference OTUs, respectively.

**
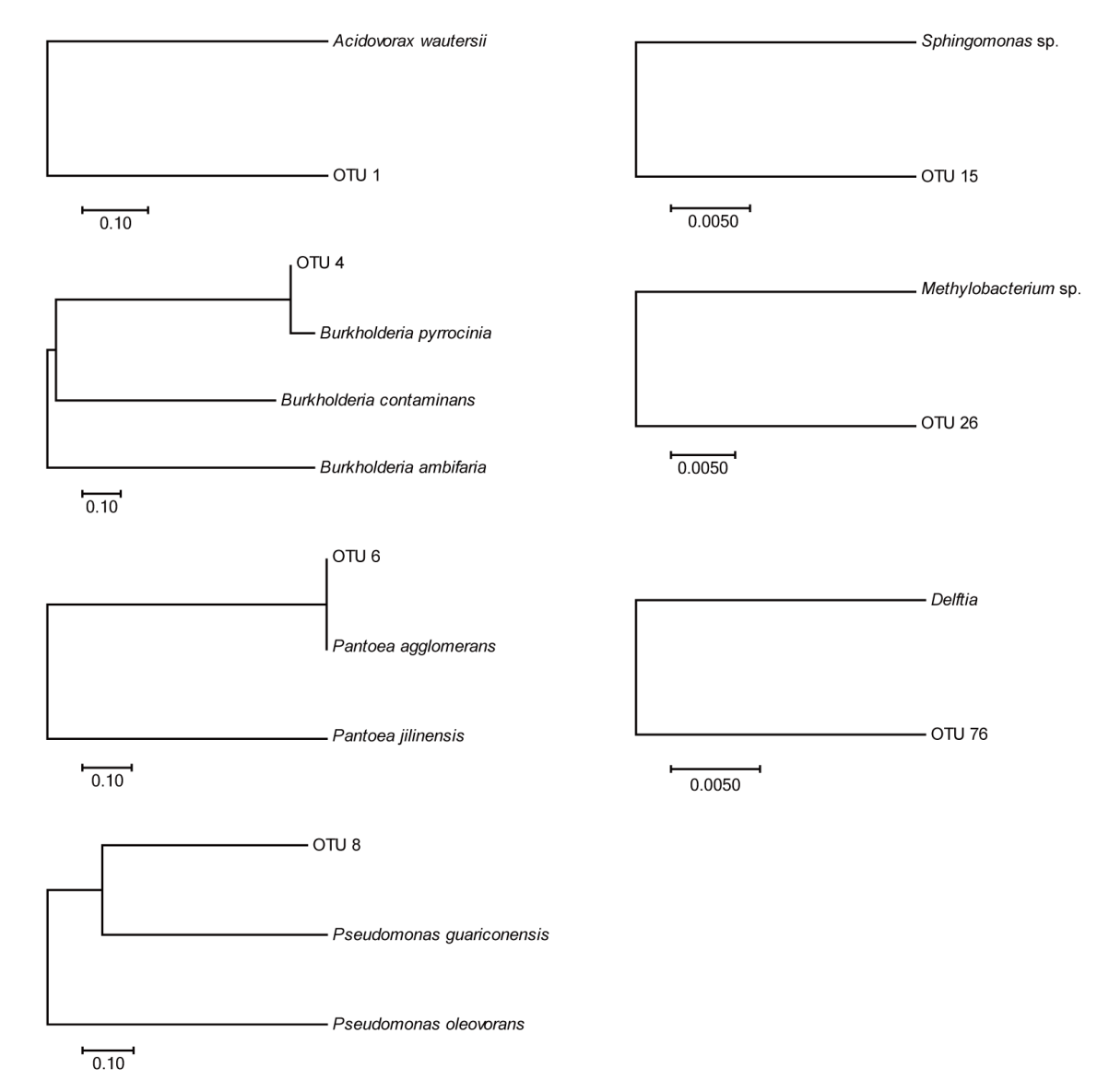
**

**Figure S3 Molecular Phylogenetic analysis by Maximum Likelihood method.** The evolutionary history was inferred by using the Maximum Likelihood method based on the Tamura-Nei model. The tree is drawn to scale, with branch lengths measured in the number of substitutions per site. Evolutionary analyses were conducted in MEGA7.

**
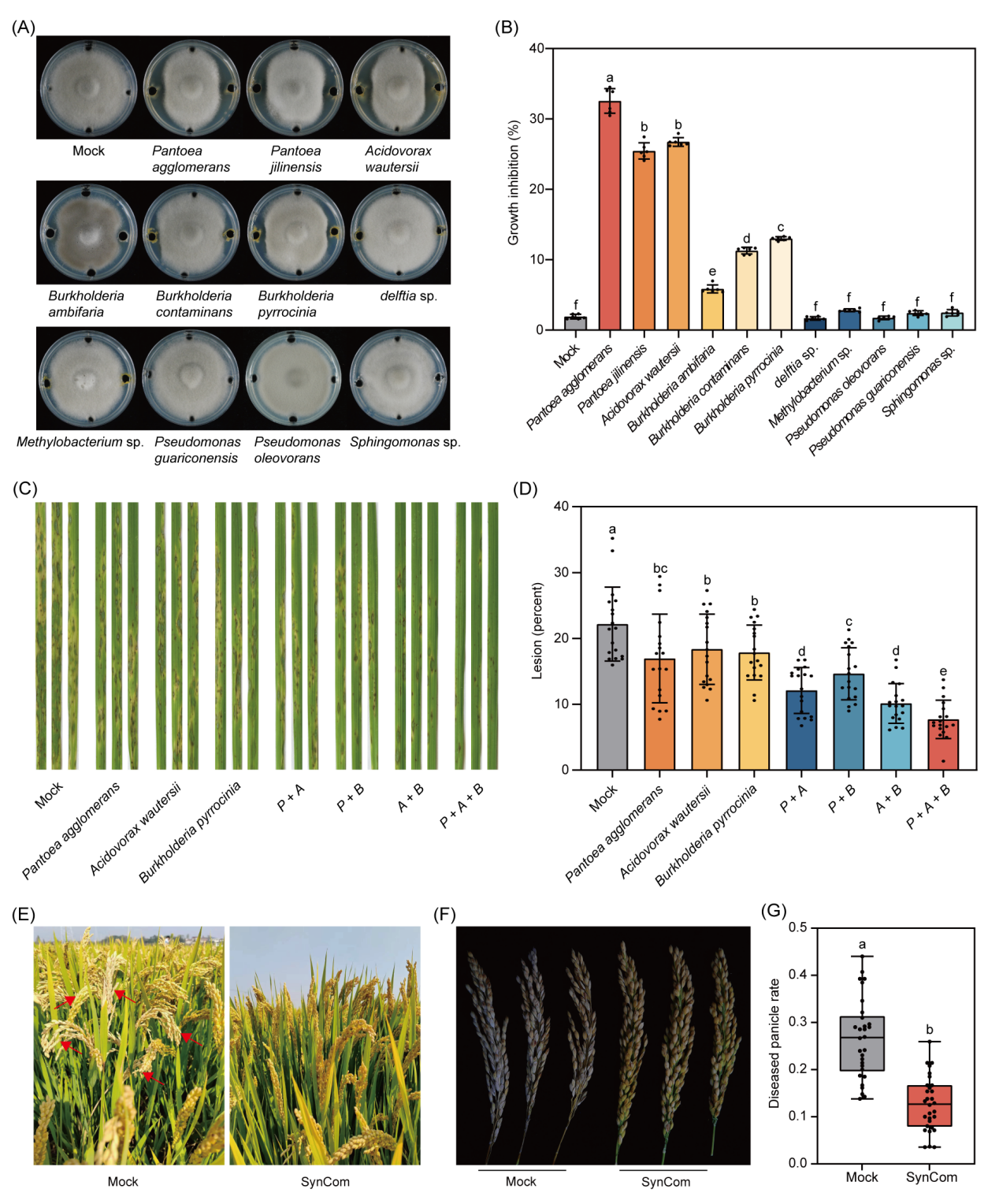
**

**Figure S4 Antagonistic and biocontrol activity of identified bacterial strain inhibiting rice fungal pathogen *M. oryzae*.** (A) Antagonistic activity of screening strain towards *M. oryzae* in co-cultured assays, using untreated strain as control (Mock). (B) The growth of inhibition of screening strain towards *M. oryzae*. The experiments were repeated three times independently with similar results. (C) Disease lesions areas on greenhouse-grown rice inoculated with antagonistic isolates and *M. oryzae.* Lesion areas were determined at 15 dpi (n = 6 independent leaves, means ± SD). (D) The rate of disease lesion caused by *M. oryzae* was altered in rice leaves treated with different antagonistic isolates. Different letters indicate significant differences at *p* < 0.05, as determined by one-way ANOVA with Tukey’s multiple comparisons test. The experiments were repeated three times independently with similar results. (E) Field test of the disease suppression activity of the SynCom (P + A + B). (F) Representative images of *M. oryzae* symptoms in rice panicles. (G) The diseased panicle rate treated with the SynCom. Values are means ± SD (n = 30). Different letters indicate significant differences at *p* < 0.05, as determined by unpaired Student’s t-test (two-tailed).

**
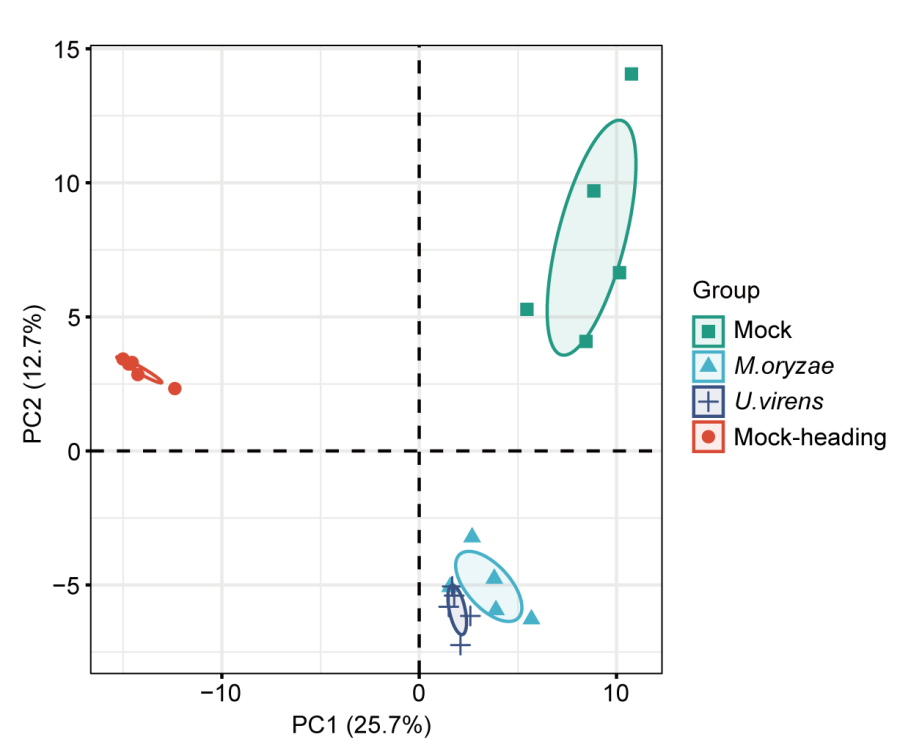
**

**Figure S5 Principal Component Analysis (PCA) analysis of bacterial communities.** Based on Bray-Curtis distances calculated from the relative abundance of different OTUs.
